# Analysis of Inflexibility and Eating Disorders According to the Theory of Control by Justifications and Immediate Consequences (TJC)

**DOI:** 10.3390/nu18061011

**Published:** 2026-03-23

**Authors:** Carla Juliane Martins Rodrigues, Olavo de Faria Galvão, Luiz Carlos de Albuquerque

**Affiliations:** 1 Graduate Program in Neuroscience and Behavior, Behavior Theory and Research Center, Federal University of Pará, Belém 66075-110, Brazil or cmartinsbehavior@gmail.com (C.J.M.R.); ofgalvao@gmail.com (O.d.F.G.); 2 Graduate Program in Experimental Psychology, Institute of Psychology, University of São Paulo (USP), São Paulo 05508-030, Brazil

**Keywords:** eating behavior, inflexibility, flexibility, rule-governed behavior, control by justification

## Abstract

Objectives: This study investigated whether vegetarian and non-vegetarian university students exhibit distinct responses to questionnaires assessing symptoms of eating disorders and inflexibility to change. Methods: Participants completed a demographic questionnaire, the Disordered Eating Attitudes Scale, and the Behavioral Inflexibility Scale. Data were analyzed using descriptive and inferential statistics, including relative risk (RR), 95% confidence intervals (95% CI), and *p*-values. Results: Results indicated that vegetarian students presented a higher risk of disturbed eating attitudes compared to omnivores (RR = 1.17; 95% CI = 0.53–2.54; *p* = 0.69), though this difference was not statistically significant. In contrast, female students presented a significantly higher risk of disturbed eating attitudes than male students (RR = 2.72; 95% CI = 1.07–6.8; *p* = 0.02). No statistically significant differences were observed for race/ethnicity, or field of study in relation to disturbed eating attitudes. Regarding behavioral inflexibility, no significant differences were found between vegetarians and omnivores (RR = 1.74; 95% CI = 0.60–4.98; *p* = 0.29) or between female and male students (RR = 1.16; 95% CI = 0.42–3.33; *p* = 0.78). Conclusions: Additionally, participants characterized by higher behavioral inflexibility tended to exhibit more disturbed eating attitudes, highlighting the association between behavioral rigidity and eating-related patterns. The results are analyzed according to the TJC.

## 1. Introduction

The Theory of Control by Justifications and Immediate Consequences (TJC) seeks to identify the variables involved in the establishment and maintenance of individuals’ broad behavioral repertoires. Specifically, TJC aims to identify the variables that lead individuals to perceive as they do, hold particular beliefs and values, make specific choices, develop certain preferences and desires, engage in particular actions, think and feel in certain ways, acquire knowledge, and acquire other behavioral repertoires [1]. In this context, TJC also seeks to identify the variables involved in the establishment and maintenance of eating behavior.

Reward and punishment centers of the brain

Eating behavior depends on multiple variables. In addition to nutrient deprivation, it is influenced by cultural, social, and economic factors [1,2]. Human behavior, including eating behavior, depends on physiological variables and the functions of reward and punishment circuits in the neural system. When behaviors are followed by rewarding consequences, they are more likely to be maintained, whereas behaviors associated with punitive consequences tend to be reduced. Therefore, reward and punishment circuits in the brain play a central role in the regulation and maintenance of individuals’ activities [3,4,5].

Distinctions between the TJC and other theories

However, according to the TJC, assumptions based on physiological data are not sufficient on their own to explain individuals’ broad repertoires, including eating behavior. To do so, it is also necessary to consider the roles of environmental variables. Behavior analysis differs from other approaches in psychology mainly because it is characterized by performing a functional analysis of behavior. TJC performs a functional analysis of behavior, but unlike other behavior-analytic theories, it distinguishes among rules, justifications, and immediate consequences. In addition, TJC differentiates between behavior maintained by immediate consequences and behavior maintained by justifications [1,6,7].

Distinction between verbal and nonverbal environments 

According to TJC, humans differ from other animals mainly because humans are exposed to a verbal environment, whereas other animals are exposed to nonverbal environments. The verbal environment consists of (a) antecedent verbal stimuli, functioning as rules and justifications, and (b) consequent verbal stimuli, functioning as immediate verbal consequences. The nonverbal environment, on the other hand, consists of antecedent and consequent nonverbal stimuli that do not function as rules, justifications, or immediate verbal consequences. Therefore, within a verbal environment, individuals can learn through both justifications and immediate consequences. In contrast, within a nonverbal environment, learning occurs exclusively through immediate nonverbal consequences. These differences in learning processes are fundamental to explaining the primary distinctions between the learning repertoires of humans and those of other animals. Thus, the terms “rules,” “justifications,” and “immediate consequences” are used to refer to variables that control behavior and to tools for analyzing behavior [1,6,8].

Distinctions between rules, justifications, and immediate consequences

According to TJC, immediate consequences are nonverbal and verbal stimuli produced by behavior that can maintain that behavior altering the function of antecedent and consequent stimuli, regardless of justifications [1,6]. The types of immediate consequences are positive reinforcement, negative reinforcement, punishment, and extinction [9,10]. For example, a nonverbal immediate consequence produced by the behavior of eating broccoli is the taste of the ingested food. A verbal immediate consequence produced by this behavior could be the following comment: “Very good!”. If the behavior of eating broccoli recurs, the immediate consequence is said to have strengthened its maintenance. Conversely, if the behavior of eating broccoli does not recur, the immediate consequence is considered not to have strengthened its maintenance. Thus, immediate consequences can alter the likelihood that a behavior will be maintained in the future [1,6].

Rules are verbal antecedent stimuli that describe behavior and can serve multiple functions. Their primary functions are to evoke behavior and to alter the functions of antecedent and consequent stimuli. Examples of rules include: “Prefer natural foods,” and “Avoid ultra-processed foods,” [1,6].

Justifications are antecedent verbal stimuli that indicate present, historical, and future events and that can maintain behavior and alter the functions of antecedent and consequent stimuli, regardless of immediate consequences. For example, after being exposed to the rule: “Avoid ultra-processed foods,” the listener may be exposed to justifications such as: “Frequent consumption of ultra-processed foods can contribute to the development of diseases such as obesity” [1,6].

Justifications can be Type 1 (JT1)—indicators of future events; Type 2 (JT2)—indicators of antecedent approval; Type 3 (JT3)—indicators of the listener’s trust in the speaker; Type 4 (JT4)—indicators of the form of verbal stimuli; and Type 5 (JT5)—indicators of what to observe. Behavior maintained by JT1 would occur more for the benefit of the listener than for others. Behavior maintained by JT2 would occur more for the benefit of others and future generations than for the listener. Behavior maintained by JT5 would benefit both the listener and others, as well as future generations. JT3 and JT4 interfere with the control of justifications over behavior [1,6,8].

Therefore, rules can evoke behavior; however, behavior evoked by rules may or may not be maintained, depending on immediate consequences and justifications. Thus, both immediate consequences and justifications can maintain behavior. Unlike control by immediate consequences, control by justifications involves reported historical, present, and future events that function as verbal antecedent stimuli constituting the justifications themselves. In this manner, justifications perform their functions as current substitutes for the historical and future events they report, regardless of the immediate consequences produced by the behavior and independent of the space and time contiguity between the justification and the behavior it maintains [1,6].

For example, the willingness or desire to eat a particular food can be established and maintained either through a history of control by immediate consequences or through a history of control by justifications. In the first case, individuals may desire a food because, in the past, consuming it (behavior), produced pleasurable experiences (immediate consequence produced by the behavior), which they now seek to experience again. In the second case, individuals may desire a food because they have previously been exposed to justifications indicating that the food is highly palatable or because they have been exposed to justifications suggesting that the food will provide numerous health benefits [1,6].

Control by justifications also differs from control by rules. Rules indicate the behavior to be exhibited. For example: “Do this,” “Don’t do that,” “Choose this,” “Don’t choose that,” and so on. In contrast, justifications indicate why the behavior should or should not be maintained. For example, after being exposed to the rule “Try to start eating broccoli,” an individual may also be exposed to justifications such as “Broccoli is a functional food and its consumption is important because it helps reduce bad cholesterol, contributes to the proper intestinal functioning, and aids in the prevention of cancer, premature aging, thrombosis, and stroke.” Thus, rules evoke behavior, and justifications alter the likelihood that the behavior will occur and be maintained. For instance, the behavior indicated by the rule “Start doing this” may have its likelihood of occurrence and future maintenance altered by justifications such as these: “And you’ll end up getting fat,” or “And you’ll end up losing weight” [1,6,8].

Distinction between behavior maintained by justifications and behavior maintained by immediate consequences

The distinctions examined in this study may be clarified by considering that, according to TJC, behavior can be maintained through (1) antecedent approval, indicated by justifications, and (2) consequent approval, indicated by immediate consequences. The difference is that, in antecedent approval or disapproval indicated by justifications, the stimuli (such as criticism, praise, indications of admiration, rejection, behavior considered correct or incorrect, good or bad) are antecedent and, therefore, presented before the occurrence of the behavior to be maintained. For example, following the rule “Eat broccoli,” a justification may be added, such as “I will be very happy if you start eating broccoli.”

In contrast, in consequent approval or disapproval indicated by immediate consequences, these stimuli (such as criticism, praise, indications of admiration, rejection, and behavior considered correct or incorrect, good or bad) function as consequent events and are presented after the occurrence of the behavior. For instance, after the listener eats broccoli, the following immediate consequence is presented: “I was very happy that you ate broccoli.” In both cases, the behavior of eating broccoli may be maintained; however, maintenance through approval should be attributed to the effects of justifications in the former case and to the effects of immediate consequences in the latter. According to this proposition, behavior specified by a rule may be maintained by justifications, by immediate consequences, or by the interaction between these variables [1,6].

Combined justifications

In the verbal environment, justifications can be contacted in audio, video, and text, such as in conversations, films, and books. In general, a given justification, especially JT4, is not made available in isolation but rather in combination with other justifications [1,6]. For example, a speaker may present the rule: “Don’t be so disobedient, try to be a more obedient person…” and add the following combined justifications: “…and you will find that by being obedient, it will be easier for you to get the things you want so much” (JT1); “By being obedient, you will see that people will be prouder of you and like you more” (JT2); “If you become more obedient, your life will change for the better and the people around you will be happier. I guarantee it. You can trust me” (JT1 and JT3); “That’s my advice to you” (JT4); “Take so-and-so as an example, he has always been obedient and now he is doing very well. Now look what happened to such-and-such, he has always been disobedient and now he is in jail” (JT5) [11].

Concurrent justifications

Justifications are often in competition. An individual may come into contact with justifications that favor one choice while simultaneously encountering competing justifications that support different choices [1,6].

For example, an individual may be exposed to the rule: “Start eating healthily” and justifications such as: “A healthy diet, especially when combined with regular exercise and water intake, can contribute to weight control, strengthen the immune system, prevent premature aging, prevent the occurrence of chronic non-communicable diseases (such as circulatory system diseases, diabetes, cancer, and chronic respiratory disease), and increase the likelihood of a long and healthy life.” [1,6].

However, an individual may also contact concurrent justifications that favor unhealthy eating. For example, justifications that indicate that eating is one of life’s great pleasures and that eating behavior should produce feelings of pleasure and eliminate aversive feelings of deprivation and anxiety. Driven by such justifications, individuals may begin to feel free and satisfied to the extent that they choose to eat that produce immediate reinforcing consequences. The interaction between these justifications and immediate consequences can make this eating pattern very persistent. However, an individual may also come into contact with justifications indicating that adopting a healthy eating pattern has several advantages for maintaining an individual’s health [1,6].

According to TJC, the main role of concurrent justifications is to lead individuals to formulate their own justifications; that is, to evaluate the advantages and disadvantages reported in different justifications and to draw conclusions based on such evaluations. An individual’s own justifications, together with their history of control by justifications, their history of control by immediate consequences, as well as the immediate consequences and circumstantial justifications they encounter, may contribute to the direction and maintenance of their broad repertoire [1,6].

For example, an individual may be exposed to circumstantial justifications favorable to adopting a particular diet [1], such as vegetarianism [12,13]. The combined justifications for becoming a vegetarian are noteworthy because, in addition to indicating that this dietary behavior pattern can promote health, they also indicate that it can promote animal welfare, environmental preservation, and respect for religious rules [1,14]. However, while there are justifications that indicate the disadvantages of meat consumption, there are also justifications on the nutritional benefits (such as B vitamins) of moderate consumption of meat prepared in a healthy manner. Again, exposure to concurrent justifications may lead individuals to formulate their own justifications, evaluating the advantages and disadvantages indicated by circumstantial justifications and to tell themselves or others what and why they decided. Together, individuals’ history of control by justifications and their history of control by immediate consequences contribute to the direction and maintenance of eating behavior [1]. It is noteworthy that there are research findings indicating that vegetarians tend to exhibit disordered eating behaviors [15,16,17].

Similarly, individuals, especially young women, are often exposed to justifications indicating that their physical appearance is considered a relevant variable for them to be successful in life. Thus, in the verbal environment, there are justifications indicating that individuals tend to be considered successful, accepted, admired, beautiful, attractive, and desirable when they are thin. Contact with such justifications may contribute to the generation and maintenance of an individual’s desire to be thin. It may also contribute to the generation and maintenance of the fear of gaining weight or becoming overweight. It can further contribute to an individual’s pursuit of the objectives contained in the justifications, namely, to be thin and avoid weight gain. In this manner, the individual may come into contact with justifications that indicate that certain restrictive diets have the advantage of producing rapid weight loss [1,6].

At the same time, an individual may also come into contact with circumstantial justifications indicating that restrictive diets have disadvantages, such as impairing health and reducing immune function. Exposure to such concurrent justifications may contribute to the individual’s formulation of personal justifications; that is, it may lead the individual to evaluate the advantages and disadvantages of pursuing an idealized thin body and avoiding weight gain. This process may also contribute to the experience of anxiety, guilt, regret, stress, low self-esteem, and depression. Furthermore, it may increase the likelihood of contact with circumstantial justifications favorable to the adoption of behaviors classified as eating disorders, such as anorexia and bulimia [1,6].

Anorexia is characterized by the adoption of a restrictive eating pattern, possibly maintained by combined justifications that indicate that such an eating pattern will produce an idealized thin body, the extinction of fear of weight gain, and acceptance by others [1,18]. There are circumstantial justifications in the verbal environment that indicate that those who manage to remain anorexic are strong, special individuals, and examples to be followed [19]. However, at the same time, there are also justifications indicating that maintaining anorexia may lead to illness and even death [20]. Bulimia, on the other hand, is characterized by food restriction, episodes of binge eating followed by purging, and feelings of regret, maintained by combined justifications indicating that such behavior can prevent weight gain and maintain a body approved by others. There are justifications indicating that bulimia is approved [19]. Nevertheless, there are also justifications indicating that bulimia will produce gastrointestinal complications, as well as other health problems, and therefore should not be considered an example to be followed [20].

Thus, a critical characteristic of individuals with anorexia or bulimia is that they have an inflexible repertoire; that is, a repertoire that is resistant to changes required by environmental variables. For example, they have an inflexible concern with body shape and weight. Some individuals may inflexibly believe that they are overweight, despite evidence indicating otherwise—that their body is excessively thin—and that an ideal body is a future situation that is not clearly achievable. This characteristic is possibly maintained by combined justifications that indicate the advantages of having an idealized body and the disadvantages of not having such a body, and of seeking to have such a body as a future event that is not clearly attainable [1,18].

Individual differences

Flexibility and inflexibility have been considered personality traits and used to explain individual differences between individuals [21]. Thus, flexibility would be a predictive factor that an individual would tend not to follow rules, and inflexibility would be a predictive factor that an individual would tend to follow rules [22]. Personality differences have been used to explain individual differences related to eating practices [23,24,25]. Vegetarianism, as a personality trait, would be a predictor of the type of food an individual tends to consume. Anorexia, on the other hand, would be a predictor that an individual adopts a restrictive diet, is afraid of gaining weight, and seeks an idealized body [1].

An alternative explanation is that individual differences in characteristic behavioral repertoires, or personality differences, arise from variations in individuals’ histories. Vegetarians may have a history of exposure to justifications that emphasize the advantages of vegetarianism, whereas individuals with anorexia may have a history of exposure to justifications that indicate the perceived advantages of acting and thinking in ways characteristic of that condition. Furthermore, experimental results suggest that the possible effects of personal histories on determining individual differences depend, in part, on current circumstantial variables. Thus, the broad repertoires characteristic of an individual, or personality traits, are established over the course of an individual’s history of exposure to justifications and immediate consequences [1,7].

The combined effects of these histories, along with the effects of immediate consequences and circumstantial justifications can alter, restrict, or expand an individual’s repertoires [1,7]. For example, if vegetarians and omnivores were asked to give their opinion on an ideal diet, they would likely report different opinions, according to their respective histories, and these historical variables, together with circumstantial variables, may interfere with their food choices [1,7,17].

Considering this analysis, research in the field of eating behavior that compares the reports of vegetarians and omnivores students from different academic disciplines and courses on issues related to inflexibility to change and symptoms of eating disorders may contribute to the identification of both the variables involved in the maintenance of eating behavior and the defining properties of this behavior. Accordingly, the present study aimed to assess whether vegetarians and omnivores students enrolled in health-related programs and those in other fields of study may present different response patterns in questionnaires commonly used to assess tendencies toward rule-following flexibility or inflexibility and the presence of eating disorder symptoms. To this end, university students were asked to complete two questionnaires in the following order: (1) the Disordered Eating Attitudes Scale [26]; and (2) the Rigidity Scale [21], adapted by Jonas [27].

## 2. Materials and Methods

### 2.1. Type of Study

This is a quantitative, cross-sectional, and analytical study conducted during the months of May and June 2022.

### 2.2. Participants

The participants were undergraduate students from the Adventist University Center of São Paulo (UNASP), located in the city of São Paulo, SP, Brazil. Participation was voluntary, and students were invited through oral announcements made in classrooms over a period of approximately 30 days. Students were eligible for inclusion if they (a) were between 18 and 30 years of age; (b) were regularly enrolled at the institution; (c) agreed to participate in the study; and (d) completed the questionnaire used as the data collection instrument. Students residing in university dormitories were excluded because the options offered in the university restaurant are exclusively ovo-lacto-vegetarian, which could influence participants’ reports.

The sample size calculation based on a target population of 2485 college students [28,29] assumed a 5% confidence level (α = 0.05, SD = 1.96) and a maximum allowed error of 5%, resulting in a minimum sample size of 168 individuals. Twenty percent was added to the calculated sample size to account for possible losses due to dropouts, incomplete data, or other reasons, resulting in a projected sample size of 205 individuals.

The final sample of participants included in the study was 205 individuals. The demographic distribution of the participants was as follows: 149 females (73%) and 55 males (27%). Regarding ethnicity, the distribution was as follows: 3 Asian (1%), 100 White (49%), 2 Indigenous (1%), 63 “Pardo” (31%), and 37 Black (18%). Regarding fields of study, the participants were classified as 114 students from health programs (56%), 70 from humanities programs (34%), and 21 from exact sciences programs (10%). Regarding academic year, they were as follows: 73 in the 1st–2nd term (36%), 40 in the 3rd–4th term (19.5%), 33 in the 5–6th term (16%), 44 in the 7–8th term (21.5%), and 15 in the 9–10th term (7%). Regarding diet, the data were as follows: 139 omnivores (68%) and 66 vegetarians (32%); 2 participants with behavioral flexibility (1%), 187 as average (91%), and 16 as inflexible (8%); and 33 presented disturbed eating attitudes (16%), whereas 172 did not (84%).

### 2.3. Procedures and Data Collection

The study began after approval by the Research Ethics Committee of the Tropical Medicine Center of the Federal University of Pará, Brazil, under opinion number 5.330.546, on 4 April 2022, CAAE 54208621.0.0000.5172, and by the Research Ethics Committee of the UNASP, Brazil, under opinion number 5.407.205, on 13 May 2022, CAAE 54208621.0.3001.5377.

Data collection took place in a computer room according to the availability of the Informatics Laboratory and the University Library. The questionnaires were displayed on the computer screen. Students were informed about the study through posted notices and oral announcements in classrooms over a 30-day period. The questionnaires were administered in a single session lasting 20 min (on average) in a private room with one computer for each participant. Participants were exposed to three stages, respectively: (1) reading and signing the informed consent to participate in the study and completing a characterization form; (2) completing the Eating Disorder Attitudes Scale; and (3) completing the Rigidity Scale.

### 2.4. Data Collection Instruments

Three data collection instruments were used: (1) a sociodemographic data form developed by the researchers; (2) the Disordered Eating Attitudes Scale; and (3) the Rigidity Scale. These instruments were presented electronically via Google Forms^®^.

The sociodemographic data form included questions about sex (female, male, or “preferred not to state”), age, undergraduate course, academic year, ethnicity and classification of dietary pattern adopted (omnivorous or vegetarian). Subsequently, the Disordered Eating Attitudes Scale [26] and the Rigidity Scale [21], adapted by Jonas [27], were applied.

Disordered eating behaviors, which include disordered eating thoughts and feelings, were assessed using the *Disordered Eating Attitudes Scale (DEAS-s)*, developed and validated in Brazil (Escala de Atitudes Alimentares Transtornadas–EAAT in Portuguese). The present study used an updated version of the *DEAS-s*, also developed and validated in Brazil, with 17 self-report items [26], with some modifications to its original structure.

The questions were organized on a Likert scale or in a dichotomous format. For dichotomous items it was attributed 5 points for yes and 1 point for no, 5 for often and 1 for rarely. For Likert-type items, the response categories it was attributed 4 for always, 3 for often, 2 for sometimes, and 1 for rarely/never. Another type of scoring was used in question 7: 1 point for “return to normal eating”; 3 points for “consider that they have lost control and continue to eat even more or decide to go on some type of diet to compensate”; and 5 points for “use some form of compensation, such as physical activity, vomiting, laxatives, or diuretics”. Higher or equal to 1.5 scores are considered indicative of an eating disorder [26].

The Rigidity Scale developed by Rehfisch [21] is validated and is based on the Minnesota Multiphasic Personality Inventory (MMPI) and the California Personality Inventory [22]. This scale aims to identify self-reports of flexible and inflexible behaviors generated by histories of exposure to immediate consequences and differential justifications for following and not following rules, which could contribute to producing individuals who are more or less rule-followers than others [7,22]. The scale is a questionnaire with 39 true/false items. There is a template for evaluating responses, and one point is added to each response according to the model. Self-reports in the range of 0 to 12 correct answers (27% or less of the total possible correct answers) were considered self-reports indicative of flexibility. Self-reports in the range of 29 to 39 correct answers (75% or more of the total possible correct answers) were considered self-reports indicative of inflexibility to change. Self-reports in the range of 13 to 28 were not classified as either flexible or inflexible. The Rigidity Scale was used as it allows identifying individuals who tend to follow rules and individuals who tend not to follow rules based on their pre-experimental histories; that is, their classifications as inflexible and flexible, inferred from their responses to the Rehfisch questionnaire [21]. It was also used to make the data from the present study comparable to data from related studies that used the same scale [2,7,22,27,30,31].

### 2.5. Data Processing

The classification of participants’ dietary patterns was based on their self-reported responses in the electronic form. Participants who self-reported that they avoided consuming animal meat were classified as vegetarians. Participants who self-reported that they allowed the consumption of food, including animal meat, were classified as omnivores. Throughout data collection, responses were monitored to assess the proportion of vegetarian and non-vegetarian participants, ensuring a minimally balanced distribution between groups according to the sampling plan.

Following this step, and in line with criteria established in the literature, participants were analytically grouped into two main categories: vegetarians and non-vegetarians (omnivores). This classification was applied exclusively during data processing, after data collection was completed.

### 2.6. Data Analysis

The data were collected using Google Forms and exported to a spreadsheet for analysis (Microsoft Office 2013 for Windows, Microsoft Corporation, Washington, DC, USA). Descriptive and inferential statistical analyses were then performed.

The categories were organized to facilitate analysis, with undergraduate courses named by area: health, humanities, and exact sciences; and religions were Adventist, Catholic, no religion, or other.

Numerical data were presented as mean and standard deviation when they exhibited a normal distribution or as median and confidence interval when the data were non-parametric. To assess the distribution of the numerical variables, we conducted tests of normality and visually inspected the data. Categorical variables were summarized using absolute and relative frequencies. Variables showing signs of redundancy were removed from the final models. We also examined whether the variability of the data was consistent across groups. All analyses were performed using appropriate statistical software, adopting a significance level of α = 0.05.

A paired chi-square test of independence, without continuity correction, was performed to assess the relationship between the following nominal variables: (1) Rigidity Classification (flexibility or inflexibility); (2) *DEAS-s* Classification (1, 2, 3, or >4), in this study, scores 5, 6, and 7 were included in score 4; (3) Sex (male or female); (4) Dietary pattern (omnivorous or vegetarian); and (5) Course (exact sciences, humanities, or health-related). Results were considered statistically significant when *p* < 0.05, the *odds ratio* greater than 1, and the 95% confidence interval. The statistical power of the chi-square test of independence was estimated based on the effect size, sample size (n), significance level (α = 0.05), and the test’s degrees of freedom.

Additionally, a multiple linear regression model was constructed including the significant correlations between variables (stepwise method), to identify which ones were related to the scores on the “Rigidity” and “DEAS-s” instruments. In all cases, the established significance level α was 5% (*p* < 0.05).

A correlation could be predicted from the scores on the Rigidity scale and the *DEAS-s* scale by linear regression, b = 0.10, t (203) = 18.92, *p* < 0.001, with the rejection of heteroscedasticity (change in variance between the initial and final segments) by a two-tailed Goldfeld–Quandt test, QG (101, 100) = 1.23, *p* = 0.304.

## 3. Results

The prevalence of reports of disturbed eating attitudes was 16% (28 women, 5 men) and inflexibility to change was 8% (12 women and 4 men). Only 2 (1%) of the 205 participants exhibited flexibility to change. Therefore, it was not possible to perform statistical analysis of the flexible participants. The remaining 187 participants (91%) obtained an average result on the Rigidity Scale; therefore, they cannot be classified as flexible or inflexible (Table 1).

The relevant results were those that were statistically significant in the three measures of analysis: odds ratio, confidence interval, and *p*-value.

Regarding reports of eating disorder symptoms, female participants were 2.72 times more likely (95% CI 1.07 to 6.8) to report such symptoms than male participants (Table 2).

Regarding the association between dietary patterns and disturbed eating attitudes, omnivores were more frequently classified as 1, whereas vegetarians were more often classified in categories 2 or 3 on the DEAS-s scale. These findings suggest that higher scores on the scale indicate a greater tendency to present symptoms of disturbed eating attitudes. However, only scores above 4 were considered indicative of these symptoms.

Multiple linear regression was used to predict the influence of the other study variables on the DEAS-s score. Only the variables “Inflexibility” and “Age” were significant at the 5% level in the model (the others were excluded due to co-linearity): [F (2, 201) = 9.507; *p* < 0.001; R^2^ = 0.086]. These variables explain 8.6% of the variability in the DEAS-s score, and the equation describing this relationship is: DEAS-s score = 28.366 + 0.611 (Inflexibility) − 0.524 (Age). That is, for each 1-point increase in *DEAS-s*, there is a 0.611 increase in the inflexibility variable and a 0.524 decrease in age. Therefore, “Inflexibility” (β = 0.243; t = 3.593; *p* < 0.001) and “Age” (β = −0.178; t = −2.636; *p* = 0.009) are predictors of *DEAS-s*.

The influence of the other variables on the ‘Inflexibility’ score was analyzed. Only the variable ‘DEAS-s’ remained significant at the 5% level in the model, with the other ones excluded due to co-linearity: [F (1, 202) = 11.723; *p* = 0.004; R^2^ = 0.055]. The DEAS-s variable explains 5.5% of the variability in the Inflexibility score, as described by the following equation: Inflexibility Score = 18.928 + 0.093 × (DEAS-s). This means that for each additional point in the DEAS-s score, an increase of 0.093 points in the Inflexibility score is expected. Therefore, DEAS-s (β = 0.234; t = 3.424; *p* < 0.001) is a significant predictor of Inflexibility.

**Figure 1:**
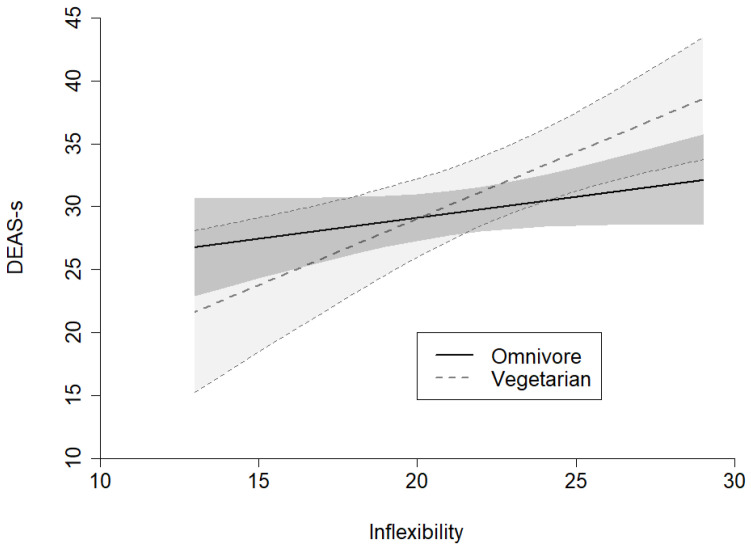
Analysis of the effect of the dependent variable DEAS-s moderated by dietary pattern and the independent variable Inflexibility.

The results suggest an interaction between the dependent variable (DEAS-s) and the independent variable (inflexibility) moderated by dietary pattern. There is a statistically significant effect of inflexibility on DEAS-s for the vegetarian group (*p* = 0.0495). The effect of inflexibility depends on the dietary pattern specified by the regression lines (Figure 1).

## 4. Discussion

The present study aims to assess whether vegetarians and omnivores college students enrolled in health-related program courses and other areas, may present different response patterns on questionnaires assessing whether an individual has a flexible or inflexible tendency to follow rules and whether or not they present eating disorder symptoms.

In the present study, similar to the results found in similar studies on eating behavior [2,32,33], few participants (i.e., only 16%) reported symptoms indicative of eating disorders; and, similar to the results found in studies in the area of rule-governed behavior [6,10,34,35], few participants (i.e., only 8%) reported inflexibility, and only 2 of 205 participants demonstrated flexibility to change. Therefore, the majority of the sample (84%) did not report eating disorder symptoms or inflexibility to change.

Unlike the study that identified that nutrition students were more likely to present eating disorder symptoms than students from other health-related courses (biomedical sciences, physical education, nursing, physiotherapy, medicine, and occupational therapy), the present study did not find such differences [36]. One possible explanation for not finding significant differences between the reports of students from different courses on eating behavior could be that currently, the justifications for adopting a healthy diet are not only available in nutrition courses, but also in audio, video, and text formats, in the media, on social networks, in conversations at work, at home, and in universities [1].

However, similar to the results found in the literature [16,17,37], the results from this study showed that vegetarians were more likely to have eating disorder symptoms than omnivores. It might be said that, unlike omnivores, vegetarians may tend to present eating disorder symptoms because they are commonly exposed to concurrent justifications about the advantages and disadvantages of eating meat. Exposure to such concurrent justifications, in certain circumstances, may contribute to certain disorders in vegetarians [1].

Also similar to the results found in the literature [36,38,39], the results of the present study indicated that eating disorder symptoms were more prevalently found in female students than in male students. It is possible that these differences occur because, unlike men, women are exposed to justifications that indicate that they will be more accepted and admired if they are thin. In general, such justifications may contribute to young women wanting to have an idealized thin body, to avoid obesity, and to be accepted. They may also contribute to individuals adopting restrictive eating patterns that could prevent weight gain. However, at the same time, the individual may also be exposed to justifications that indicate that such restrictive eating patterns may be harmful to health [1].

The results of the current study suggest a correlation between inflexibility to change and disturbed eating attitudes. From this perspective, inflexible individuals tend to exhibit disturbed eating attitudes, and individuals with disturbed eating attitudes tend to exhibit inflexibility to change.

The results of the present study are consistent with those reported by Pinto et al. [30], who found that participants classified as inflexible individuals tended to follow rules, whereas those classified as flexible individuals tended not to follow them. In that study, participants were previously exposed to justifications indicating that the behavior specified by the rule would earn points that could be exchanged for money; however, the points were actually added to the behavior of not following the rule. These results indicate that the participants’ pre-experimental histories, that is, their classifications as inflexible and flexible individuals, inferred from their responses to the Rehfisch [21] questionnaire, may contribute, in part, to predicting the occurrence of differences between performances observed in the same situation [1,7].

Nevertheless, there are also results showing that both flexible and inflexible participants failed to follow rules when the justifications indicated that the behavior specified by the rule would prevent the loss of points exchangeable for money, and what actually prevented the loss of points in the experimental situation was the behavior of not following rules [31]. Together, these results from the studies by Pinto et al. [30,31] suggest that in Pinto et al. (2006), it was the circumstantial variables present in the experimental situation that, in relation to the study by Pinto et al. [31], probably favored the differences between the performances of flexible and inflexible participants [1,7].

These experimental results suggest that the possible effects of personal stories in determining individual differences depend, in part, on circumstantial variables, such as the immediate consequences and justifications that an individual may encounter in certain circumstances [1,6,7]. This assumption is also supported by results from applied studies. For instance, there is evidence suggesting that the repertoires of individuals with eating disorder symptoms can be altered by psychotherapies such as cognitive behavioral therapy [20,40,41].

According to the cognitivist model (model adopted in a large part of data analyses in neurophysiological studies and studies on individual differences), it is an individual’s perceptions and interpretations of events, rather than circumstantial variables alone that determine an individual’s repertoires [20,42,43]. In contrast, according to TJC, an individual’s broad repertoires—including eating behavior—are established and maintained by the combined effects of historical and circumstantial variables. Therefore, an individual’s repertoires on perceptions, interpretations, thoughts, beliefs, among others, are established and maintained, to some extent, by historical and circumstantial variables, such as immediate consequences as well as historical and circumstantial justifications. For instance, in psychotherapy, some of the questions, comments, and explanations presented by the therapist to the client may function as justifications that alter the client’s perceptions, interpretations, thoughts, beliefs, and other repertoires. Thus, an individual’s perceptions, interpretations, thoughts, and beliefs may interfere with their lifestyle, including his/her eating behavior [1].

According to this proposition, combined religious justifications may indicate that a person will have divine protection and will go to heaven after death if they come to value and believe in what is prescribed by the religion. In contrast, combined educational justifications may indicate that a person will be approved in courses, receive scholarships, prizes, and distinctions, expand their knowledge, obtain a good job, gain recognition, and be considered “immortal”, provided that they attempt to do what education approves of. Similarly, combined nutritional justifications may indicate that a person will have a long and healthy life if they attempt to do what is approved by experts in the field. Such justifications, together with combined political, economic, institutional, cultural, and other justifications, would contribute to the formation of people’s broad repertoires. These broad repertoires, functioning as individuals’ own justifications, could interfere with their subsequent behavior under certain circumstances [1]. Future research may contribute to clarifying these propositions.

Self-report bias is a notable factor, as some participants may have responded under the influence of what they believe to be expected or socially desirable. However, the results obtained do not show significant discrepancies compared to previous studies in the literature, suggesting that the findings are consistent. Another limitation concerns gender diversity. Participants were categorized according to the most reported genders—female and male. Although this approach homogenizes part of the sample and does not encompass the full diversity of gender identities, it allows for a consistent initial analysis and provides a starting point for future research that may expand the investigated categories.

## 5. Conclusions and Implications

The findings and implications of this study suggest that (a) college students in health-related fields do not significantly differ from students in other fields of study in their tendency to exhibit eating disorder symptoms; (b) vegetarian university students are more likely to exhibit eating disorder symptoms than omnivorous students; (c) female students are more likely to exhibit eating disorder symptoms than male students; and (d) inflexible participants tend to exhibit disturbed eating attitudes, and participants with disturbed eating attitudes tend to be inflexible.

The results of the present study therefore suggest that inflexibility to change and disturbed eating attitudes are correlated. The results of basic research and applied studies suggest that inflexibility to change depends, in part, on circumstantial variables. Taken together, these results suggest that the repertoires of an individual showing inflexibility are resistant to change and difficult to alter; however, this does not imply that they are immutable, since such repertoires can be altered depending, in part, on circumstantial variables to which the individual is exposed. This analysis is consistent with the TJC proposition that an individual’s repertoires, including eating behavior, are largely established and maintained by circumstantial and historical variables, such as immediate consequences and circumstantial and historical justifications.

## Figures and Tables

**Table 1:** Characterization profile of the sample (n = 205).

Variables	Categories	n	%
Gender/Identity	Female	149	73.0
	Male	55	27.0
Race/ethnicity	Yellow	3	1.0
	White	100	49.0
	Indigenous	2	1.0
	Brown	63	31.0
	Black	37	18.0
Field of knowledge	Health	114	56.0
	Humanities	70	34.0
	Exact sciences	21	10.0
Academic Term	First to second	73	36.0
	Third to fourth	40	19.5
	Fifth to sixth	33	16.0
	Seventh to eighth	44	21.5
	Nineth to tenth	15	7.0
Eating Pattern	Omnivorous	139	68.0
	Vegetarian	66	32.0
Flexibility	Flexible	2	1.0
	Average	187	91.0
	Inflexible	16	8.0
Disturbed eating attitudes	Yes	33	16.0
	No	172	84.0
Total		205	100.0

**Table 2:** Odds ratio, confidence interval, and *p*-value of reports of eating behaviors and inflexibility to change.

Categories	Compared Variables	Relative Risk *	95% CI **	*p* ***
Disturbed eating attitude	Vegetarians vs. omnivores	1.17 *	0.53–2.54	0.69
	Female vs. male	2.72 *	1.07–6.8	0.02 ***
	White vs. black and “pardo”	1.46 *	0.67–3.18	0.32
	Health-related programs vs. humanities and exact sciences programs	1.46 *	0.67–3.18	0.32
	Vegetarians vs. omnivores	1.74 *	0.60–4.98	0.29
Inflexibility to change	Female vs. male	1.16 *	0.42–3.33	0.78
	White vs. black and “pardo”	0.86	0.30–2.4	0.78

* If the result is higher than one unit, the chance of A being higher than B is accepted. ** Values higher than one unit show no difference between the groups studied. *** *p* < 0.05 indicates a statistically significant difference.

## Data Availability

The data presented in this study are not publicly available due to ethical restrictions related to the potential identification of participants. However, selected data that do not compromise participant privacy may be made available upon reasonable request to the authors, provided that the intended use complies with ethical principles and confidentiality guidelines. All data are securely stored in the author’s personal research database, in line with MDPI’s data preservation policies.
